# Parasite contamination of berries: Risk, occurrence, and approaches for mitigation

**DOI:** 10.1016/j.fawpar.2018.04.002

**Published:** 2018-04-21

**Authors:** Tamirat Tefera, Kristoffer R. Tysnes, Kjersti Selstad Utaaker, Lucy J. Robertson

**Affiliations:** Laboratory of Parasitology, Department of Food Safety and Infection Biology, Faculty of Veterinary Medicine, Norwegian University of Life Sciences, Adamstuen Campus, P.O. Box 369 center, 0102 Oslo, Norway

**Keywords:** Berry, Parasite, Contamination, Control, Outbreak

## Abstract

Fresh fruits and vegetables, including berries, are essential components of a healthy diet and are relevant in the prevention of chronic non-communicable diseases such as cancer and heart disease. Associations between diet and health are becoming an increasing focus of consumers, and, in response, consumption of fresh berries has been increasing rapidly in recent decades. However, increased consumption of berries may be associated with an increased risk of acquiring foodborne infections, including parasites. In this review, we describe how parasite contamination of berries may occur at several points on the farm-to-fork pathway, starting from the use of contaminated water for irrigation and pesticide application, and contact with animal and human faeces during cultivation, through contaminated harvesting equipment, and including unhygienic practices of berry pickers in the production field or others handling berries prior to consumption. Parasite transmission stages tend to be robust and therefore likely to survive from contamination in the field, through the various stages of harvesting, packaging, and sale, until consumption. We describe outbreaks of parasitic disease associated with consumption of berries – so far only described for *Cyclospora* and *Trypanosoma cruzi*, both of which are briefly introduced – but also show from survey data summarised in this review that sporadic infections or undetected outbreaks associated with contaminated berries may also occur. In addition, we describe methods for assessing whether berries are contaminated with parasite transmission stages, with emphasis on the challenges associated with analysing this particular matrix. Emphasis on current possibilities for mitigation and control are addressed; avoidance of contamination and implementation of good management practices and a hazard analysis and critical control points (HACCP) approach are essential.

## Highlights

• Berry consumption, production, and export are rising steeply.
• Berries can act as transmission vehicles for various parasites.
• Outbreaks are documented; removal and inactivation measures are limited.
• Standardization of laboratory detection methods is warranted.
• Hazard analysis and critical control points (HACCP) approach to limit contamination.

## 1 Introduction

Foodborne diseases are major public health concerns throughout the world, irrespective of the wealth index of countries. Although preventable, foodborne diseases cause significant morbidities, ranging from mild to severe, and in some cases resulting in mortality. There is also an associated deleterious effect on the socio-economy; in addition to the suffering from disease and costs incurred for treatment, the absence of affected people from the workplace has a negative consequence on the economy and productivity among people with sub-clinical infections might also be reduced (Torgerson, 2013). Furthermore, outbreaks of disease associated with fresh produce not only harm that industry but shake the confidence of consumers in a food group that is of importance in a healthy diet.

Although the combined effect of foodborne parasitic diseases globally was estimated to be the loss of almost 12 million disability-adjusted life years (DALYs) (Torgerson et al., 2015), relatively scant attention has been paid to the parasitic foodborne diseases. This is probably due to various factors, including: i) many parasites have complex lifecycles and thus investigations can be complicated and difficult; ii) the diseases caused by foodborne parasites often have long incubation periods, and thus source attribution of most foodborne parasitic diseases is difficult (FAO/WHO, 2014); iii) for many foodborne parasites, standard laboratory analyses are lacking or poor (Robertson, 2014); iv) doctors seldom consider the possibility of foodborne illness having a parasite aetiology.

**Fig. 1:**
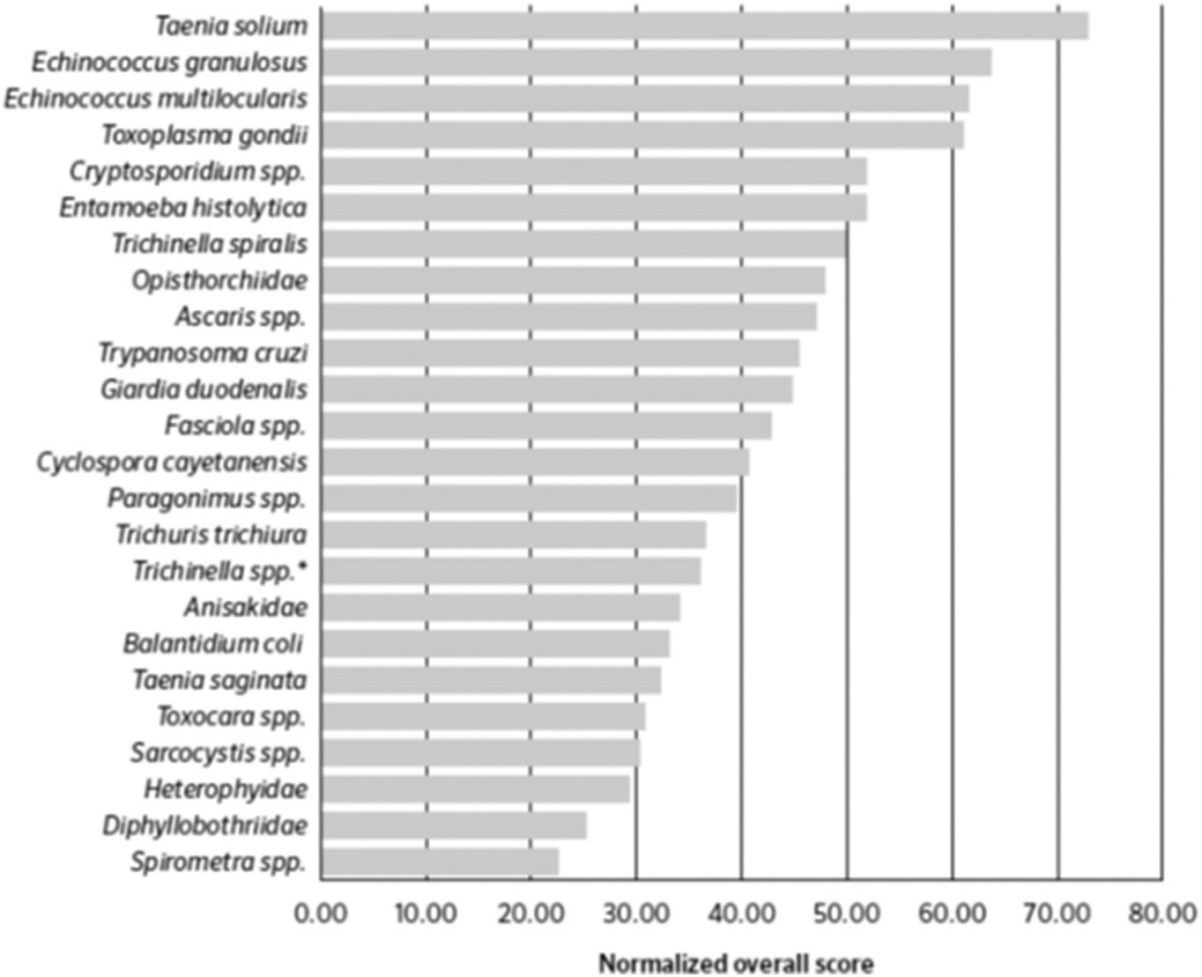
Global ranking of foodborne parasites.

A panel of experts appointed by Food and Agriculture Organization and World Health Organization (FAO/WHO) published a multicriteria-based ranking of foodborne parasites, which considered the most important parasites transmitted by food on a global scale. Of the 24 parasites included in this exercise, the top 15 parasites, in descending order, were *Taenia solium*, *Echinococcus granulosus*, *Echinococcus multilocularis*, *Toxoplasma gondii*, *Cryptosporidium* spp., *Entamoeba histolytica*, *Trichinella spiralis*, Opisthorchiidae, *Ascaris* spp., *Trypanosoma cruzi*, *Giardia duodenalis*, *Fasciola* spp., *Cyclospora cayetanensis*, *Paragonimus* spp., and *Trichuris trichiura* (Fig. 1) (FAO/WHO, 2014). With the exception of *Paragonimus* spp., Opisthorchiidae, and *Trichinella spiralis*, all these “top 15” parasites have the potential to be transmitted via fresh produce (FAO/WHO, 2014).

(Source: FAO/WHO, 2014).

A similar exercise conducted to prioritize foodborne parasites in Europe gave a slightly different picture (Bouwknegt et al., 2018), with the top 15 parasites being *Echinococcus multilocularis*, *Toxoplasma gondii*, *Trichinella spiralis*, *Echinococcus granulosus*, *Cryptosporidium* spp., *Trichinella* spp. other than *T*. *spiralis*, *Giardia duodenalis*, Anisakidae, *Toxocara* spp., *Taenia solium*, *Ascaris* spp., Opisthorchiidae, *Taenia saginata*, *Entamoeba histolytica*, *Diphyllobothrium* spp. However, again, the majority of these can be transmitted by fresh produce.

In this review, we take a specific area of the fresh produce industry, berries, and consider their importance as a vehicle for transmission of parasites. The reason we believe that berries are of particular importance is that consumption of this type of fresh produce has increased enormously in recent years, and berries are frequently imported from countries where some parasitic infections are endemic that may be considered rare or unusual in importing countries. Furthermore, berries are often consumed raw and, indeed, some types of berries are difficult to wash prior to consumption without affecting their quality. Thus, contaminating parasites are difficult to remove and may be viable and infectious when consumed.

Having provided further details on the growth in berry consumption, we consider not only how berries may become contaminated, but also adhesion of parasites to berries and their survival on this matrix. Outbreaks associated with consumption of contaminated berries are then described, economic impacts associated with such impacts, methods to detect parasite contamination of berries and the results of surveys, and, finally, potential approaches to control are discussed.

## 2 Berry production

From a botanical perspective, a berry is a stoneless fruit that is produced from one flower with a single ovary; such a definition excludes some fruit that most consumers would consider to be berries (e.g., strawberries, raspberries), but includes produce such as aubergines, cucumbers, and bananas. For the purposes of this review, we do not use the botanical definition, but consider berries as fruits derived from a variety of plants and which are characterized by a high surface-weight ratio and the entire fruit, including seed, can be consumed in a succulent form (Codex Alimentarius, 2000). These tend to be relatively soft fleshed, small diameter pieces, and lack a peel or inner core, such as, for example, strawberries, raspberries, blackberries, and blueberries.

One characteristic of this type of fresh produce is that some species can be harvested from the wild and also cultivated. In addition, although berries may be grown on a large-scale, it is also common that they are grown on small production sites; such situations may be more vulnerable to pathogen contamination due to less advanced infrastructure, and reduced ability to follow the principles of good agricultural practice (GAP) and good handling practice (GHP) (Ganpat et al., 2014; European Commission, 2006).

Over the past few decades, there has been a steady increase in the demand for fruit and fruit-based products, as consumers seek out healthier dietary options. In particular, over and above many other fresh produce, berries are considered to be one of the best dietary sources of bioactive compounds that have important antioxidant properties, with associated health effects such as protective effects against some cancers and cardiovascular disorders (Skrovankova et al., 2015). In addition, their supportive effects on lipid profiles, fasting plasma glucose, and blood pressure levels are thought to be beneficial towards metabolic disorders such as diabetes (Skrovankova et al., 2015).

The UK is one of the biggest markets for berry sales, and over the last decade, sales here alone have risen by 132%; in the U.S., raspberry and blueberry consumption has risen by 411% and 475%, respectively (Barro, 2014). In addition to the increase in fresh berry consumption in many countries, we are also consuming increasing numbers of berries in other forms, such as smoothies and shakes. The compound annual growth rate of the smoothie market is envisaged to exceed 7% from 2016 to 2020 (Statista, 2015).

The enhanced consumer demand for berries has increased their cultivation. Raspberry production has benefited from this trend, although production of other fresh berries, such as blueberries and blackberries, has also grown tremendously during the last few decades. Many of the countries in which berry production is on the rise are those with warm climates, to ensure year-round production, and often have cheap labour costs. Such tropical and sub-tropical countries may not only be endemic for pathogens that are unusual or rare in the importing countries but also may have a less developed infrastructure for avoiding contamination of the berries during production. For example, Morocco, which is endemic for cystic echinococcosis (Chebli et al., 2017), exports blueberries to various European countries where this disease does not occur, and, similarly, *Cyclospora* is endemic in various tropical and sub-tropical countries, including Morocco (Chacin-Bonilla, 2017), from which berries are exported.

**Fig. 2:**
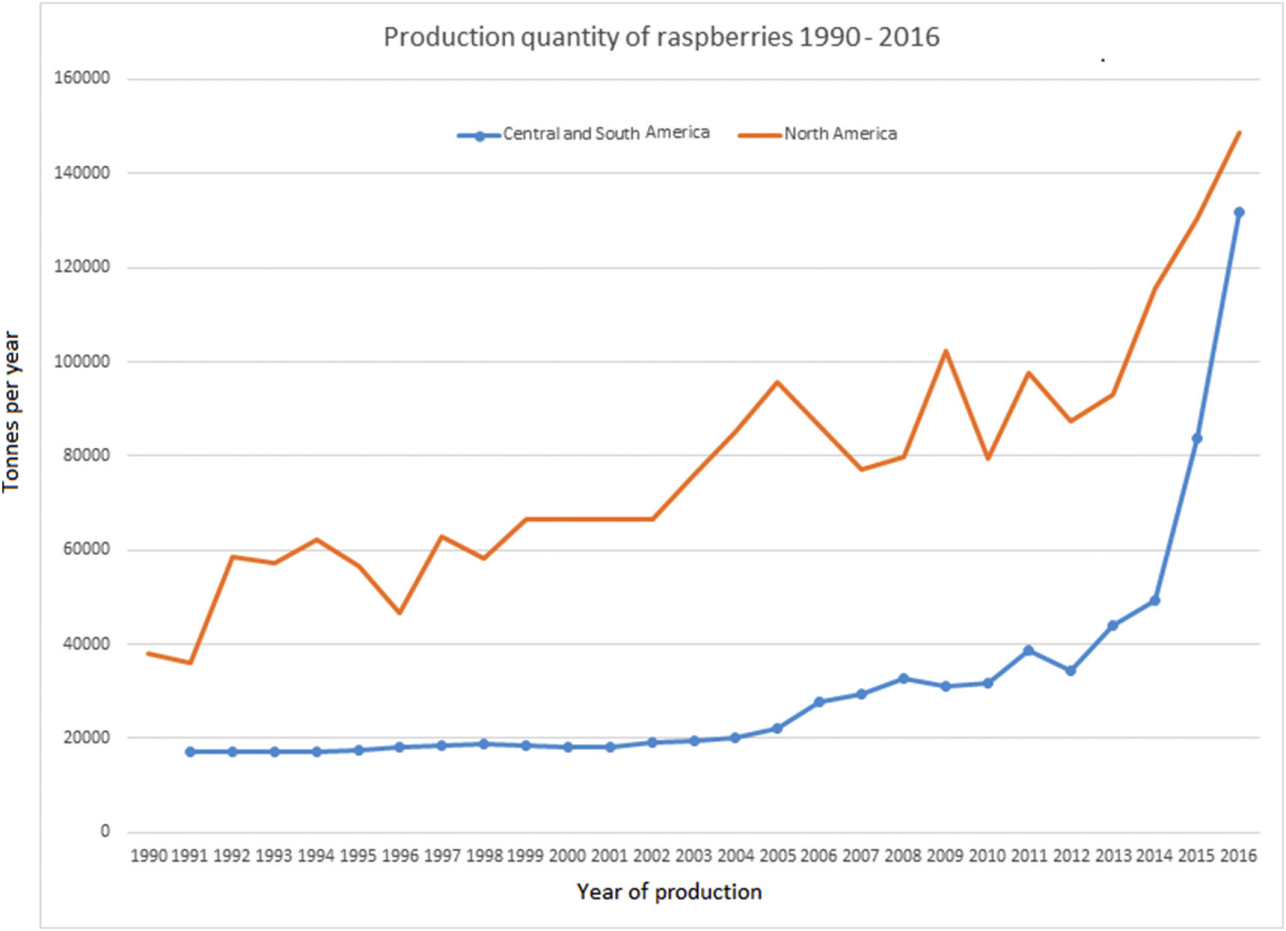
Comparison of the production quantities of raspberries in North America with production in Central and South America between 1990 and 2016 (FAOSTAT)*. *Aggregates, may include official, semi-official, estimated or calculated data.

**Fig. 3:**
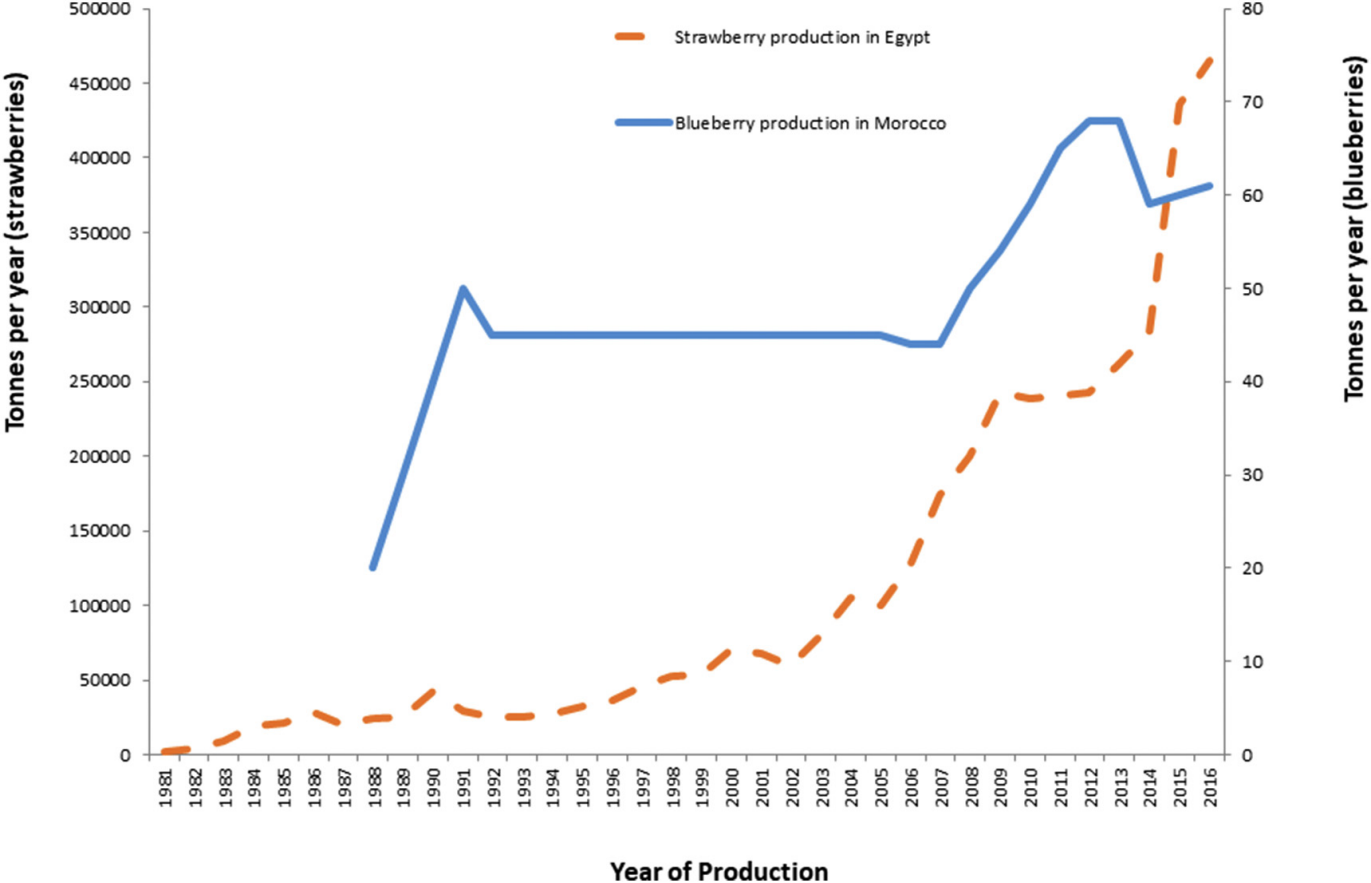
Production trend of blueberries in Morocco and strawberries in Egypt during 1981–2015.

An increase in production of raspberries has occurred in Central and South America since the early 2000s, in conjunction with the rise in demand in Northern America. In Mexico, raspberries are not a traditional agricultural product, but the demand for an out-of-season supply to markets in the U.S. and Canada, where the demand for fresh berries all year round continues to rise, has been the main driver for the development of this production in these areas (Kempler and Hall, 2013). The increased production quantities of raspberries in North America and Central and South America over time are presented in Fig. 2, while the increase in production of blueberries and strawberries in Morocco and Egypt, respectively, are shown in Fig. 3.

(Data from FAOSTAT).

## 3 Routes of parasite contamination of berries

**Fig. 4:**
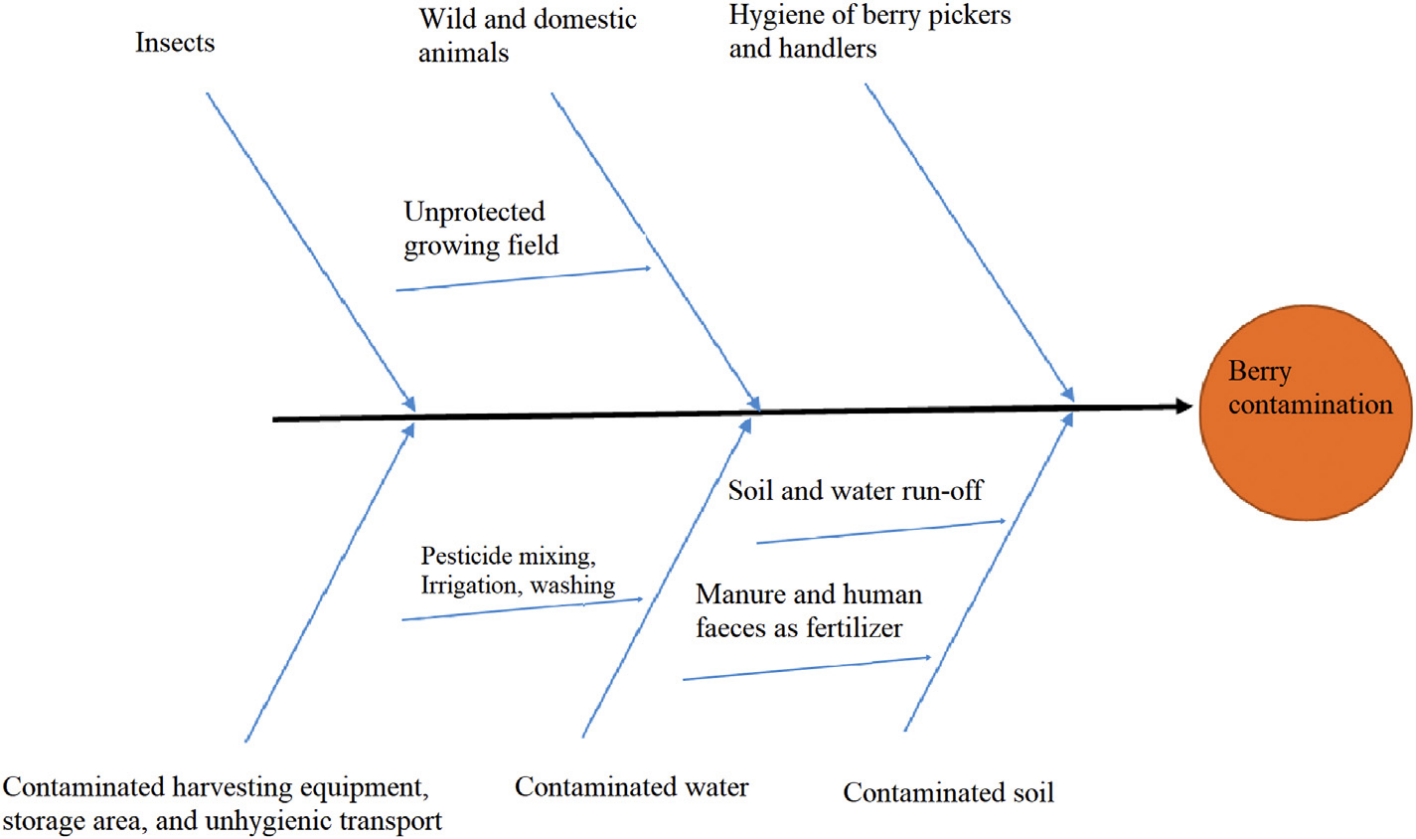
Ishikawa representation of the routes of parasite contamination of berries.

Berries, like any fresh produce, can become contaminated with pathogens at several points along the farm-to-fork pathway (Fig. 4). The routes of contamination can be considered in two groups: during production in the field (including harvest), and after harvesting. In the field, potential sources of contamination include the use of contaminated water for irrigation or spraying, and for mixing pesticides and insecticides. Common water treatment processes, such as chlorination, are not effective at inactivating parasite transmission stages, and not all irrigation water is necessarily treated anyway; WHO has recently changed their guidelines for irrigation water and there are now no definitive values for microbiological guidelines. Instead, irrigation water safety should be based upon risk assessment as recommended in WHO documents and water guidelines should rely on standards within respective countries, which may vary greatly (Drechsel and Keraita, 2010).

A study on contamination of berries with enteric viruses demonstrated that irrigation water was an important and relevant source of contamination (Maunula et al., 2013). The berry-growing field may also be contaminated from the soil and water run-off due to location; municipal waste disposal or treatment sites in the proximity of berry growing field should be considered. Faecal contamination from wild and domestic animals could also occur in areas where the farm is not protected (Beuchat, 1996). The use of animal and human waste as fertilizer could be an additional source of parasitic contamination, either directly or potentially with transport via insects. The hygienic status of farm workers who are involved as hand pickers may affect the parasitological safety of berries during harvest (Fig. 4). The cyclosporiasis outbreak linked to Guatemalan raspberries in 1996 and 1997 in the U.S. might have been partly associated with contamination from infected farmworkers (Bern et al., 1999).

Post-harvest, the equipment used for sorting and packing is a potential route of parasitic contamination, and storage and transport conditions might also affect the parasitological safety of the berries (Beuchat and Ryu, 1997; FDA, 1998; Oger et al., 2001). During processing, if the berries are washed, this may result in contamination either from the water itself or by cross-contamination from contaminated batches. Again, during processing berries may be extensively handled, and the enteric virus study (Maunula et al., 2013) also indicated this could be a route of contamination. Furthermore, how the berries are handled and presented at the sales point may result in contamination from other customers, as has been indicated from studies on fresh produce in India (Utaaker et al., 2017a).

## 4 Adherence and survival of parasites on berries

For berries to present a risk to human health due to contamination by parasite transmission stages, these stages must adhere to the berries and survive until consumption by a suitable host. Regarding adherence, many parasite transmission stages are known to be sticky; *Ascaris* eggs, for example, are well known for this, although the nature of their adherence has not been fully characterised (Quilès et al., 2006). Taeniid eggs, such as those of *T*. *solium* or *Echinococcus* spp., are also known to have a sticky coat. Furthermore, one study showed that none of the elution methods commonly used for detection purposes were able to completely remove *Cryptosporidium* from apples (Macarisin et al., 2010). Using scanning electron microscopy in this study, the oocysts were seen attached to the apple surface by a filamentous matrix, but it was not possible to tell whether the matrix originated from the parasite itself or from the apple. *Cyclospora* oocysts are considered to be even more sticky than *Cryptosporidium* oocysts, due to specific adhesins (Ortega and Shields, 2015).

In addition, the nature of the berries may assist in attachment of the parasites. It was shown that *T*. *gondii* oocyst can attach to and remain infective after inoculation on both blueberries and raspberries (Kniel et al., 2002). With its hairy projections on the surface, it was no surprise that raspberries were a more effective vehicle for transmission to mice in the bioassay than blueberries, which have smoother surfaces.

It is known that many parasite infectious stages can survive for long periods in the environment and that the survival rate may increase under cold and humid storage conditions (Lelu et al., 2012; Robertson and Gjerde, 2006; Utaaker et al., 2017b). In order to keep berries fresh, they are usually transported from production sites to sales points in cool conditions, which are also ideal for maintaining the infectivity of any adherent parasites. In an experimental contamination of apples with *Cryptosporidium parvum* oocysts, mice became infected by oocysts after storage for up to 4 weeks on the apples (Macarisin et al., 2010). A recent report indicated that refrigerated conditions contributed to the increased survival of *G*. *duodenalis* cysts compared with storage at room temperature (Utaaker et al., 2017b). It was noted that about half of the *Giardia* cysts did not survive for one day when kept at room temperature. Interestingly, *Cryptosporidium* spp. oocysts were shown to be more robust and there was no significant difference in their survival between the different storage conditions. As it has been shown that storage under cool and damp conditions may prolong the survival of some parasites, storage conditions should be considered carefully when considering the risk of human infection and keeping fresh produce in good condition.

A study on the survival of *Toxocara* eggs showed an interesting feature in that the parasite eggs ceased development during the winter season while retaining their viability, and then resumed their development in the spring (Kazanina, 2014). *Ascaris* eggs have also been shown to resist harsh environmental conditions including freezing temperatures (−9 °C to −12 °C), and the eggs have been shown to resume embryonation once incubated at room temperature (WHO, 2004). Their resistance to harsh environmental conditions enhances parasite survival, and thus the probability of infection from consuming contaminated berries.

## 5 Outbreaks of parasitic disease associated with contaminated berries

Fresh produce remains the number one vehicle for most outbreaks of foodborne illnesses, according to the Centre for Science in the Public Interest (CSPI, 2015). Most outbreaks linked to berries have been viral diseases, particularly those caused by norovirus and hepatitis A, although *Shigella sonnei* has also been transmitted via berries (Tavoschi et al., 2015). Among parasites, the only documented transmission via berries has been for the intestinal protozoan parasite *Cyclospora cayetanensis* (Palumbo et al., 2013a) and for the systemic protozoan parasite *Trypanosoma cruzi*, for which transmission has been via açaí berries, or, more commonly, drinks made from these berries (de Noya et al., 2015).

Although berries may have also been associated with other outbreaks, these have not been identified. The relatively prolonged incubation period between infection and symptoms for nearly all parasites – ranging from days (*Cryptosporidium* spp.) to months or even years (*Echinococcus* spp.) - means that identifying an associated food vehicle is very difficult (Eckert and Deplazes, 2004). Even for *Cryptosporidium*, it is unlikely that samples for testing would be available, having been either discarded or consumed, by the time symptoms occurred. However, the fact that multiple outbreaks of cyclosporiasis associated with berries have been detected compared with none for cryptosporidiosis, indicates that there may be a special association between this parasite and berries. This may reflect the areas of geographical endemicity, but could also be because *Cyclospora* oocysts simply survive better on fresh produce. For *T*. *cruzi* infections associated with açaí berries, or drinks made from açaí, this could be a reflection of the association between triatomine vectors and this fruit, as well as being associated with the locations and conditions of making products from these berries.

### 5.1 Cyclosporiasis

*Cyclospora cayetanensis* is an apicomplexan, coccidian protozoan that causes a self-limiting diarrhoeal disease known as cyclosporiasis. The parasite oocysts, which autofluoresce under UV excitation, are approximately spherical with size ranging between 8 and 10 μm in diameter. *C*. *cayetanensis* has a simple direct life cycle with humans as its only major known host. The oocysts are shed in faeces unsporulated and hence are not immediately infectious; it takes days or weeks for the oocysts to become sporulated in the environment at temperatures between 22 and 32 °C (https://www.cdc.gov/dpdx/cyclosporiasis/index.html).

The first reported cases of cyclosporiasis (in retrospect) were reported in the late 1970s, during which it was mostly associated with traveller's diarrhoea. It was initially reported as coccidian-like or cyanobacterium-like-body (CLB), but was fully characterized in the early 1990s, and during this decade at least 11 definite and probable outbreaks affecting at least 3600 persons were documented. These numbers were, and are still, unprecedented in the experience with foodborne outbreaks of other protozoan parasites, such as *Giardia* and *Cryptosporidium* (Strausbaugh and Herwaldt, 2000). It was not until the 1995 cyclosporiasis outbreak in the U.S. and Canada this parasite really captured the attention of researchers and physicians (Ortega and Sanchez, 2010).

In the U.S., outbreaks of cyclosporiasis have been reported since the mid-1990s and most are linked to fresh produce such as raspberries, basil, and cilantro. According to the CDC summary report for the years 2000–2015, a median of two outbreaks occurred every year, with a median of 21 cases per outbreak (ranging from 3 to 582 cases) were reported (CDC, 2015). Some of the outbreaks that are specifically associated with consumption of berries are summarised in Table 1.

**Table 1:** Documented outbreaks of cyclosporiasis linked to consumption of berries and berry products.

Year (month)	Place	No. of cases	Type of berry	Ref.
1995	Florida, U.S.	38	Raspberries	Huang et al., 1995
1996	U.S. and Canada	1465	Raspberries	Herwaldt et al., 1997
1996	Boston, U.S.	57	Berry dessert	Fleming et al., 1998
1996	U.S. (Multiple states) and Ontario, Canada	850	Raspberries	Palumbo et al., 2013a
1997	U.S cruise ship (departure Florida)	220	Raspberries	CDC, 1997
1997	U.S. (Multiple states) and Ontario, Canada	1012	Raspberries	Palumbo et al., 2013a
1998	Ontario, Canada	192	Raspberries	Palumbo et al., 2013a
1998	Ontario, Canada	221	Raspberries (garnish)	CDC, 1998
1999	Florida, U.S.	94	Most likely berries in a fruit salad.	Strausbaugh and Herwaldt, 2000
1999	Ontario, Canada	104	Blackberries, raspberries, strawberries	Anonymous, 2000
May 2000	Georgia, U.S.	19	Raspberries and or blackberries (suspected)	CDC, 2015
Jun 2000	Pennsylvania, U.S.	54	Raspberries	CDC, 2015
Dec. 2001-Jan 2002	Vermont, U.S.	22	Raspberries (likely)	CDC, 2015
Jul 2008	California, U.S.	45	Raspberries and/or blackberries (likely)	CDC, 2015
2009	Connecticut, U.S.	8	Raspberry and blackberry	CDC, 2015

The outbreak of cyclosporiasis in 1996 (May–July) in two provinces of Canada (Ontario and Quebec) and 20 U.S. states and District of Columbia affected 1465 people. The outbreak was traced to fresh raspberries imported from Guatemala (Sewell and Farber, 2001). After reports of a cyclosporiasis outbreak happening again as a result of Guatemalan raspberries in 1997, the U.S. banned the import in 1998.

After the outbreak, the U.S. Food and Drug Administration (FDA), Centers for Disease Control and Prevention (CDC), Health Canada and the Canadian Food Inspection Agency provided advice and assistance in developing food safety routines. Guatemala proposed a Hazard Analysis Critical Control Point (HACCP) programme, but as the methods of detection of *Cyclospora* were considered too insensitive at that time, testing for the presence of parasites on the berries was excluded from this programme. In spring 1999, FDA decided that Guatemalan producers had done everything possible to ensure food safety with the Model Plan of Excellence (MPE) - a mandatory joint programme developed by the Guatemalan Berry Commission and the government of Guatemala - and allowed entry of raspberries produced under MPE standards. One year after the implementation of the MPE programme, in 2000, two outbreaks were traced to a single farm in Guatemala. This farm was then excluded from the programme, and since then no further outbreaks associated with Guatemalan berries have been reported (Gordon, 2016).

In the UK, investigation of a cyclosporiasis outbreak showed a link to travel to Mexico and possible consumption of berries while there. A total of 79 cases (55 from England, 21 from Scotland, and 3 from Wales) were suspected of cyclosporiasis between June 1 and September 22, 2015 and 43 of them confirmed by a reference laboratory. Food histories for 45 cases were available and 43 had consumed fruit or berries; specifically, 9 cases had consumed strawberries or raspberries (Nichols et al., 2015).

### 5.2 Chagas disease

*Trypanosoma cruzi* is a protozoan parasite in the trypanosomatidae family that causes American trypanosomiasis, also known as Chagas disease. The parasite has a complex lifecycle that involves humans (or other reservoir hosts, such as opossums) and triatomine bugs. Usually, the triatomines transmit the parasite through their faeces defecated during a blood meal. *T*. *cruzi* is distributed throughout the Latin American countries, especially in the Amazon basin (Ruiz-Guevara et al., 2015). In addition to its vector-borne transmission, *T*. *cruzi* is also associated with the oral route of transmission through contamination of fruit juices, such as açaí juice. This transmission route seems to be becoming more dominant in recent years, as the vector-borne transmission is controlled (Table 2).

**Table 2:** Documented outbreaks of Chagas disease linked to consumption of berries and berry products (information obtained from Ruiz-Guevara et al., 2015).

Year	Place	No. of cases	Type of berry/product
1998	Abaetetuba, Pará State, Brazil	11	Açaí
2003	Macapa, Amapá State, Brazil	10	Açaí juice
2004	Igarape da fotaleza, Amapá state, Brazil	27	Açaí juice
2004	Tefé, Amazonas state, Brazil	9	Açaí juice
2006	Belém, Pará State, Brazil	9	Açaí juice
2006	Barcarena, Pará State, Brazil	12	Açaí juice
2007	Breves and Bagre, Pará State, Brazil	25	Açaí
2010	Rio Negro, Amazonas, Brazil	17	Açaí Palm Fruit
2010	Santa Izabel do Rio Negro (Amazonas State), Brazil	21	Açaí
2014	Belen, Pará State, Brazil	10	Açaí
2015	Carauari, Amazonas State, Brazil	12	Açaí

Contamination of fruit juices with *T*. *cruzi* may occur at the point of fruit juice making, (González et al., 2015), particularly due to an attraction of infected triatomines to the lights and/or the smell of juice, and falling in. However, for açai juice, the berries themselves (which are the small, round, blackish drupes of the açai palm, *Euterpe oleracea*) seem to be intimately associated with infected triatomines. This has been explored in detail in the work of Xavier et al. (2014) who report on the investigation of cases and outbreaks of Chagas disease associated with consumption of açai juice at locations in Belém City, Brazil. This work has indicated that the infected triatomines are probably transported with the açai berries themselves, which are harvested from islands in the Guamá river where both the triatomines and *T*. *cruzi* are endemic. In describing this association between the infected vector and the açai berry, the authors (Xavier et al., 2014), suggest that this phenomenon for transmission of *T*. *cruzi* should be described as “distantiae transmission”. This indicates that contamination of the vehicle of infection occurred some distance from where the consumers became infected.

Although *T*. *cruzi* transmission is currently largely confined to South America, the impact of this disease and the potential for contaminated açai produce extending beyond its borders, mean that this parasite and route of infection may be of relevance to a wider group of consumers. It also seems as though foodborne transmission may cause more severe disease than vector-borne transmission, possibly due to higher numbers of parasites entering the circulation through the intestinal mucosa compared with the vector-borne route (Robertson et al., 2016).

## 6 Economic impacts and stakeholder decision-making

Although the berry industry is robust and growing, detection of parasite contamination of berries or the association of parasite infections, whether individual cases or, worst-case scenario, an outbreak with contaminated berries will require decision making for relevant stakeholders and will be associated with an economic impact. This impact may not only be associated with the particular supplier or grower of a contaminated product, but also with other suppliers of the same or similar products, which, from the consumers' perception, might also be contaminated. Thus, whole industries can be affected by a single outbreak event.

In June 1996, the Texas Department of Health issued a warning regarding the ongoing large outbreak of cyclosporiasis in U.S. and Canada. This warning incorrectly identified the source of the problem as being Californian strawberries, which were, at that time, in their peak production period, and resulted in losses in revenue to the growers estimated to be at around US$16 million (Calvin, 2004). By the time that CDC issued a statement correctly identifying raspberries imported from Guatemala as being the vehicles for the *Cyclospora* infection, the exporting season had just concluded, and, at that time, the growers were not particularly affected (Calvin, 2004). However, with the outbreak recurring the following year (1997), exports to the U.S. were stopped in the middle of the spring season, and a loss of US$10 million to the Guatemalan farmers has been estimated (Calvin, 2004); cumulative losses from 1996 to 2001 amounted to around US$20 million (Cruz-Castillo et al., 2006). Other expenses, not included in this estimate, should also incorporate the over 6200 h used by the FDA investigating the outbreaks (Calvin, 2004), not to mention medical costs and costs associated with missed work and school. Five farms were permitted to export to the U.S. for the 1999 spring season. Notably, there was originally eight farms to be audited by the FDA, but one decided to leave the raspberry business and two decided not to export due to production problems that followed the outbreak. This left Guatemala with only five producers and few marketing options to the U.S., as these were already filled by other competitors, such as farms in Mexico. In 1996, before the contamination problem began, the number of raspberry growers was estimated to be 85, but by 2002, only 3 remained. For many growers, the decision to leave the berry industry was based on the losses incurred as foreign demand collapsed, rather than the cost of implementing MPE (Calvin et al., 2002; Calvin et al., 2003).

In the long run, these repeated outbreaks were devastating to the Guatemalan raspberry trade, which having been close to 400 metric tonnes in 1996, was down to almost nothing within two years. However, this vacuum was quickly filled by exporters from Mexico. The size and growth rates of exports to the U.S. from Mexico and Guatemala were very similar before 1996, but whereas the Guatemalan raspberry market to the U.S. slumped to almost nothing, in Mexico the growth was exponential, reaching 500 metric tonnes by 1998, and over 1400 metric tonnes by the year 2000. Also, although only raspberries were implicated in these outbreaks, the U.S. demand for blackberries from Guatemala was also hit, with only just over 50% of blackberry growers continuing to export fruit in 2001 (Cruz-Castillo et al., 2006). Although these outbreaks occurred over two decades ago, their lessons are still relevant. Outbreaks, particularly large ones that are in the public eye through news and other media channels, and which are associated with a particular product may have a long-lasting impact on consumer perception of not only that product, but also other products that consumers consider to be associated.

Foodborne outbreaks require a specific response – to halt further spread of the infection, to determine the cause, and to implement measures to prevent continuation or recurrence of the problem – should parasite contamination of berries be detected. However, the decision-making and reaction by authorities and other stakeholders is not so straightforward. As can be seen from the *Cyclospora* outbreaks above, consumer reaction can be devastating to the berry industry if implicated, wrongly or rightly, with a pathogen, so it is important that facts are checked and in place. If contamination is detected, then questions must be addressed: are the parasites actually infectious to people (appropriate species or genotype), is there sufficient contamination that they pose a risk, and are the parasites detected viable and infectious? Unfortunately, for many parasites, these questions may not be answered using the methods available simply for detection. Although molecular technologies may be able to address the questions regarding species and genotype, and infectious dose data are available for many parasites, the question of viability and infectivity may be more difficult to address (Rousseau et al., 2018). Nevertheless, positive molecular results for parasite contamination should not be ignored, particularly as the recovery efficiency of detection methods is low.

In addition, it is necessary to know how widespread the contamination is – is it distributed widely through the particular product, or is it batch- or lot-specific? As the shelf life of fresh berries is very limited, by the time parasite contamination is detected it is likely that the implicated batch has been bought, and probably consumed, such that withdrawal of the product from the market may not make any difference, and could only cause unnecessary concern to the consumer. In such circumstances, checking further similar products (from the same producer) on the market for contamination could be warranted, as well as investigation of how the original contamination may have occurred.

In a survey of imported fresh berries on the market in Norway between 2015 and 2016, among 55 samples (strawberries, raspberries, blueberries) one strawberry sample, imported from Spain through the Netherlands, was found to be contaminated with low numbers of *Giardia* cysts (Jensvoll et al., 2017). Bacteriological analysis found no contamination with *E*. *coli*, but follow up samples from the same batch were found also to be contaminated with *Giardia* cysts, and molecular analyses identified them being Assemblage A (and therefore potentially infectious to people). However, no assessment of viability or infectivity could be made. No illness associated with the strawberries was reported, and the produce was not withdrawn from the shops (it had all been purchased or discarded by the time the analyses were completed), but detection of contamination was reported to the Rapid Alert System for Food and Feed and production site visits were made, concentrating on irrigation water (Jensvoll et al., 2017).

## 7 Methods for detection of parasites on berries

The lack of simple, robust, and validated methods for detection of foodborne parasites means the fresh produce industries are unable to perform self-monitoring for these pathogens, and are therefore dependent on external specialized laboratories for analysis of foodborne parasites (Hurst and Schuler, 1992). Although various methods have been developed for the detection of parasites on berries, they tend to be limited to particular types of parasites. In addition, they are not used routinely by the industry but tend to be associated with research projects or limited surveys. These methods are largely based on the direct identification of the parasitic stages (microscopy) or detection of the nucleic acids of the parasites (molecular techniques). Each method has its own merits and limitations. Table 3 shows an overview of analytical methods used for the detection of parasites on berries.

**Table 3:** Laboratory methods for the detection of foodborne parasites on berries and berry products.

Method	Parasite detected	Berry type	Amount of sample	Remark	Reference
IMS followed by IFAT (ISO18744:2016)	*G. duodenalis* & *Cryptosporidium* spp.	Berry fruits	30 g	Not able to differentiate viable (oo)cysts	ISO, 2016
IMS followed by IFAT	*G. duodenalis* & *Cryptosporidium* spp.		30 g	A reduced-cost version of the ISO18744:2016 (not validated for berries but it could be potential method)	Utaaker et al., 2015
LMS followed by wet mount microscopy	*C*. *cayetanensis*	Raspberries	100 g		Robertson et al., 2000
Nested PCR	*E*. *multilocularis*	strawberries, raspberries, blueberries and cowberries	0.3–0.5 kg		Lass et al., 2017
PCR	*T*. *cruzi*	Açaí pulp	30 ml		Ferreira et al., 2016
Real-time PCR	*Eimeria* used as surrogate for coccidia of public health concern	blackberries, blueberries, cranberries, raspberries strawberries	30 g of herbs and 60 g of berries	Oocyst recovery rates ranged from 4.1–12% for berries	Lalonde and Gajadhar, 2016
Real-time PCR	*C. cayetanensis*	Raspberry	50 g of raspberries		Murphy et al., 2018
Real-time PCR	*T*. *cruzi*	Açaí pulp	50 g		Mattos et al., 2017
Real-time PCR	*T*. *cruzi*	Açaí juice	30 ml		de Souza Godoi et al., 2017
PCR	*T*. *cruzi*	Açaí-based products	Varied		Ferreira et al., 2018

The first step in analysing berries for parasite contamination is to remove any potential parasites from their surfaces. Homogenization of the berries is likely to result in considerably greater amounts of debris and inhibitors, and preliminary studies (unpublished) indicate low recoveries. Different approaches have been used for the elution of parasites from berry samples subsequent to concentration or purification of parasites in the eluate.

Various detergents and buffers have been investigated for washing the berries to detach the parasite stages. The efficacy of the wash solution is affected by a number of factors such as the sample matrix, the pH, and concentration of the solution (Cook et al., 2006; Shields et al., 2012), the time post-contamination (Chandra et al., 2014), etc. After comparison of different wash solutions, 1 M glycine (pH 5.5) was found efficient to recover *Cryptosporidium* oocysts from raspberries (Cook et al., 2006). However, in another study, 0.1% Alconox was found to be the superior wash solution for removing *Cryptosporidium* and *Cyclospora* oocysts from raspberries (Shields et al., 2012).

Molecular detection of parasites from environmental samples is challenging due to the low number of parasitic contaminants and the presence of various inhibitors. This is critical because most parasites have low infectious doses, and, unlike bacteria, parasite transmission stages cannot be cultured for subsequent detection (Ortega, 2006). The difficulty in detaching the parasitic stages from the sample and the PCR inhibitors in the samples make the scenario even worse (Robertson, 2014).

Apart from the ISO method to detect *Cryptosporidium* spp. and *G*. *duodenalis* on leafy greens and berries (ISO18744:2016) and the BAM (Bacteriological Analytical Manual)-19 methods for detection of *Cyclospora* and *Cryptosporidium*, there are no other standardized methods for detection of parasites on berries and other fresh produce. The BAM-19a method (Orlandi et al., 2004) has recently been modified regarding detection of *Cyclospora* from fresh produce, and is now replaced by BAM-19b (Murphy et al., 2017). Inter-laboratory validation of the BAM-19b method has been recently published (Murphy et al., 2018). The ISO method used to detect *Giardia* and *Cryptosporidium* is based on elution of the parasites from the berries, concentration by centrifugation, and isolation by immunomagnetic separation (IMS), followed by immunofluorescence antibody testing (IFAT) for detection. IFAT does not determine the species in the case of *Cryptosporidium* and does not differentiate between the different assemblages of *G*. *duodenalis*. Thus, positive samples may be contaminated by species of *Cryptosporidium* or Assemblages of *Giardia* that are not expected to be infectious to people. Furthermore, the method is not capable of determination of the viability or infectivity of *Cryptosporidium* and *Giardia* (ISO, 2016).

The BAM-19 method is based on the detection of DNA of *Cyclospora* by PCR after elution and concentration by centrifugation. Here, again the viability of the parasites cannot be determined since the method is based on disruption of the oocysts and endpoint detection of DNA.

As IMS has not been developed for other parasites that may contaminate berries, such as *C*. *cayetanensis*, lectin magnetic separation (LMS) has been used for concentration of some parasites. LMS has been used for detection of *C*. *cayetanensis* on raspberries, and although it improved ease of detection, did not improve detection rate by wet mount microscopy (Robertson et al., 2000). Although LMS has been used for purification of *T*. *gondii* from environmental water samples prior to detection by microscopy or PCR, it has not been optimized for berries, and the non-specific nature of lectin binding may hamper its application to fresh produce (Harito et al., 2017).

Although microscopy-based techniques are commonly used for detection of some parasites, molecular techniques are important because of their sensitivity and specificity. Furthermore, comparison of DNA sequences from different isolates may be helpful in outbreak investigations. Information from DNA sequencing can be helpful for source attribution and pinpointing the vehicle of infection which could provide significant input for the policies towards control. Among the molecular methods used for detection of foodborne parasites are conventional PCR, real-time PCR (qPCR), nested PCR (nPCR), magnetic-capture PCR, loop-mediated isothermal amplification (LAMP) assays, etc. Much of the research related to developing molecular methods for berries has focused on *Cyclospora*, with raspberries being the predominant matrix considered (Jinneman et al., 1998; Murphy et al., 2018; Shields et al., 2013; Steele et al., 2003); this is presumably a reflection of the raspberry-associated outbreaks.

In one study *Eimeria* was used as a surrogate for *Cyclospora* and tested on five types of berries using real-time PCR for final detection (Lalonde and Gajadhar, 2016). Furthermore, a method for detection of *T*. *gondii* from strawberries using conventional PCR has also been reported (de Souza et al., 2016); *E*. *multilocularis* from raspberries by using nested PCR (Lass et al., 2015), and *T*. *cruzi* from açaí juice by using real-time PCR (Mattos et al., 2017). As real-time PCR is becoming a standard laboratory tool, it is expected to become more widely employed for detection of foodborne parasites from berries. It is possible to detect more than one parasite at a time using multiplex PCR with conventional and real-time methods (Hohweyer et al., 2016; Sim et al., 2017), but has not been tested for berries to date.

As monoclonal antibodies are known for their specific binding with antigens, aptamers are being utilized for various purposes such as diagnostics. Aptamers are short oligonucleotides of DNA or RNA that bind with target molecules in a specific manner. Although there has been no published study on the use of aptamers for detection of parasites on berries, it was found promising when tested on *Cryptosporidium* oocysts spiked on mango and pineapple (Iqbal et al., 2015).

Isothermal techniques are cost-effective potential alternatives to PCR, especially in developing countries. LAMP is one of the isothermal techniques and used to amplify the nucleic acid of interest at a constant temperature (e.g., 60 °C). It uses two or more sets of primers and a polymerase with high strand displacing capacity that facilitates replication of the nucleic acid. The amplification product is detected at the end by gel electrophoresis or in a real-time fashion by employing fluorophores such as SYBR green that enables detection of the amplicon at an interval of time (e.g., every minute). LAMP was used successfully for detection of *T*. *gondii* from ready-to-eat salad (Lalle et al., 2018), but has not, to our knowledge, been tested on berries. The authors stated that LAMP is more sensitive than conventional PCR. One main drawback of LAMP is that it does not allow for post-amplification sequencing, and cross-contamination can be a problem as there is post-amplification processing.

Another isothermal technique is recombinase polymerase amplification (RPA), which was developed in 2006 (Piepenburg et al., 2006) and now commercialized by TwistDx™. RPA uses a combination of enzymes such as recombinase, polymerase, and single-stranded DNA binding (SSB) protein in the cycle of nucleic acid amplification. The amplification is fast and is designed to work at temperatures between 37 and 42 °C. Besides, the specificity, sensitivity, portability, and the possibility of both end-point and real-time detection makes RPA an attractive alternative to other molecular methods (Lobato and O'Sullivan, 2018). It has been applied in the detection of various parasites such as *T*. *gondii* (Wu et al., 2017) and *Cryptosporidium* (Wu et al., 2016). Simultaneous detection of three intestinal protozoan, *Giardia*, *Cryptosporidium*, *and Entamoeba* from faecal samples was possible through lateral flow strip combined with RPA (Crannell et al., 2016). The detection of *T*. *gondii* from soil and water through lateral flow strip combined with RPA (Wu et al., 2017), indicates that RPA might be suitable for the detection of parasites from berries, but to our knowledge has not yet been tested.

The main problem with molecular techniques such as PCR is the susceptibility to PCR inhibitors that may be found in the sample matrix. This is a major concern in developing molecular methods for berries, because they are associated with specific inhibitors that can affect the method sensitivity. The inhibitors commonly encountered in berries might include polyphenols such as anthocyanin, flavonol, ellagitannin, proanthocyanidin, polysaccharides, and phenolic acids (Schrader et al., 2012). Droplet digital PCR (ddPCR), however, is considered to resist inhibitors and to provide accurate quantitative information without the need for preparation of standard curves. The main limitation of ddPCR is it is expensive. Nevertheless, it could be used to prepare an accurate standard curve that could then be used in qPCR, and has been used in this way for the enumeration of *Cryptosporidium* oocysts from faecal samples (Yang et al., 2014). Thus, considering options other than molecular techniques may be important or being prepared to consider approaches to overcome the inhibition.

Matrix-assisted laser desorption ionization time-of-flight mass spectrometry (MALDI-TOF MS) is a high-throughput analytical technique based on the detection of mass spectral fingerprints of proteins. It has many applications in different fields of science, including clinical and veterinary parasitology for detection and identification of parasites, but has not yet been used for detection of parasites on berries. However, MALDI-TOF MS has been used for characterization of the protein profile of parasites such as *Cryptosporidium* spp. (Glassmeyer et al., 2007; Magnuson et al., 2000), *Giardia* spp. (Villegas et al., 2006), and *Entamoeba* spp. (Calderaro et al., 2015), which might be helpful in future attempts for the detection of the parasites from berries.

In general, the methods found in the literature for the detection of parasites on fresh produce vary widely. The variations include the amount of sample used, the procedure used for elution of the parasites, the concentration of the parasites for spiking experiments, and the basis of the actual method. This implies that the results obtained from different laboratories cannot always be compared directly. Clearly, this calls for collaboration between researchers to develop a standard method for the different matrices, followed by intra- and inter-laboratory validation.

In conclusion, some of the methods used for detection of parasites on berries do not have a standard, validated protocol and can best be considered as research methods. A major challenge for the development of such methods lies in the requirement for methods that are suitable for routine analysis of samples, as well as being cost-effective, easy to implement, and providing robust results. This demands more investment in method development and validation. However, it is worth mentioning that a few studies have conducted inter-laboratory validation of the analytical methods (Murphy et al., 2018; Utaaker et al., 2015). Such efforts enforce the standardization of methods used for the detection of parasites across food testing laboratories.

## 8 Surveys of berries for parasitic contamination

The prevalence of parasites as contaminants of different fresh produce have been reported from several countries, but relatively few studies have included berries in the analyses, and even when berries have been included, the number of samples used in the studies is small. This indicates the need to conduct a comprehensive survey on berries to determine the prevalence and distribution of parasites. The different parasites found contaminating berries were *C*. *cayetanensis*, *E*. *multilocularis*, *Enterobius vermicularis*, *Ascaris*, *Entamoeba histolytica*, and *Giardia*.

The overall prevalence of parasites on berries as determined by various surveys and the most commonly encountered parasites are presented in Table 4.

**Table 4:** Surveys of berries and berry products for parasitic contamination.

Type of berry	Prevalence n/N (%)	Main parasite detected	Detection method	Country	Reference
Black berries	3/50 (6)	*Cryptosporidium spp.*	Light microscopy	Costa Rica	Calvo et al., 2004
Blueberries	1/3 (33.3)	*Cryptosporidium parvum*	Real-time PCR	South Korea	Hong et al., 2014
Raspberries	4/20 (20)	*Echinococcus multilocularis*	Nested PCR	Poland	Lass et al., 2015
Strawberries	2/62 (3)	*Giardia*	IMS followed by IFAT Microscopy	Norway	Robertson and Gjerde, 2001
Strawberries	9/16 (56)	*Ascaris* spp. and *Entamoeba coli*	Light microscopy	Brazil	Silva et al., 2014
Strawberries	–	No parasites detected	Light microscopy	Poland	Kłapeć and Borecka, 2012
Strawberries	61/168 (36.3)	*Ascaris* and other protozoan cysts		Mexico	Felix et al., 1996
Strawberries	–	Negative for *Toxoplasma gondii*	Real-time PCR	Poland	Lass et al., 2012
Strawberry juice	54.28%	*Cryptosporidium* spp., *Giardia* spp., *Cyclospora*	Light microscopy	Egypt	Mossallam, 2010
Açaí-based products	14/140 (10)	*T*. *cruzi*	PCR	Brazil	Ferreira et al., 2018

## 9 Critical control points for parasitic contamination of berries

Identification of the likely sources of contamination is the first step in the control of parasites on berries, and the risk should be reduced by identifying critical control points throughout the production line. Producers can use hazard analysis critical control points (HACCP) approaches to ensure their products are safe for human consumption by installing the proper tools for detection of the occurrence of contamination and indicate a corrective action whenever the contamination, occurs all the way from farm-to-fork (FDA, 1997). HACCP is a continuous process that describes appropriate ways to monitor critical control points to ensure the safety of berries, and it is based on a proactive principle of prevention of contamination rather than trying to control the contamination after it has already occurred. Many outbreaks have been linked to produce imported from counties where HACCP implementation is suboptimal, underscoring the importance of these routines for ensuring product safety (Dixon, 2016).

Risk assessments specific to berries have also been conducted. An assessment of the risk posed by *Salmonella* and norovirus on berries was conducted by EFSA in 2014 (EFSA BIOHAZ Panel, 2014). For these pathogens it was concluded that each farm environment represents a unique situation regarding risk factors that may affect the occurrence and persistence of pathogens on berries, and that food safety management systems (e.g. Good Agricultural Practices (GAP), Good Hygiene Practices (GHP) and Good Manufacturing Practices (GMP)) should be fundamental objectives for berry producers. However, for these two pathogens, the establishment of risk-based microbiological criteria for control of these pathogens was either not considered necessary due to insufficient evidence (*Salmonella*) or insufficient data (norovirus) (EFSA BIOHAZ Panel, 2014). In Canada, a risk assessment concerned directly with *Cyclospora* on imported raspberries and blackberries, mostly identified data gaps that would need to be filled in order for risk management decisions to be made (Dixon et al., 2016).

As noted in Section 8, parasitic contamination of produce can occur before or after reaching post-harvest processing facilities through various routes in the field or poor hygienic practice by food handlers. It is therefore important that the fresh produce industry considers critical points for contamination during industrial processing. Humans working in the facility can be a source of direct contamination and/or facilitate cross-contamination of the products. Educating staff on personal hygiene standards is therefore important. In addition to humans, contaminated instruments and equipment can be a source of cross-contamination, and proper guidelines for cleaning, maintenance, and monitoring must be in place.

A wide variety of food products are produced from berries for human consumption, and these vary in terms of physical and biological properties, and in the processes involved in production. These attributes are important factors to consider when establishing a HACCP regime, and different types of berry production should be scrutinized separately in order to optimise the HACCP regimes to make up for their differences.

Another important difference in production type is outdoor vs. indoor production. Berries grown outside are at greater risk of contamination from animals or the environment. If the production is based on open field growth, the producer must ensure proper fencing, or other measures, to prevent potential hosts for zoonotic parasites coming in contact with the produce. Another important production factor is the irrigation method used. Drip irrigation, and other methods that direct water at or near the root of the plants is considered safer than overhead methods of distribution, such as sprinkling, since it reduces that chance of contaminants reaching the edible parts of the plants. Plants grown outdoors can also be contaminated indirectly by water during heavy rainfall, as contaminants from the soil may be splashed onto the plants. Provision of sanitary facilities is also helpful to prevent contamination.

The Codex Alimentarius provides comprehensive guidelines for prevention and control of parasitic contamination of fresh produce, including environmental and personnel hygiene, control of the parasitic hazards, product information and consumer awareness, a training programme for workers and the instruction and supervision of personnel (FAO/WHO, 2007).

## 10 Interventions (removal and inactivation of parasites from berries)

Although washing berries with clean water may help in removing the parasites from the surface, berries tend to be packed for consumption without further washing because they are easily damaged and their quality is affected by washing (Palumbo et al., 2013b); this is especially true for raspberries.

Efforts to remove and/or inactivate the parasites on berries should be considered to ensure parasitological safety. Currently, there are no recommended standards for removal of parasite stages and more work is needed to find practical, suitable, and effective solutions.

Due to the robust nature of their transmission stages, parasites are known to resist the normal disinfection approaches commonly used in the food processing industry, and there are limitations to the extent of processing of foodstuffs that are meant to be consumed fresh. For example, *Cyclospora* and *Cryptosporidium* are extremely resistant to the normal chlorination concentration, and hyperchlorination (high concentration of chlorine) is often used to ensure the elimination of these parasites. However, consumer safety from the chemical perspective is another challenge in applying chemical disinfectants to berries to kill the parasites. The efficacy of gaseous chlorine dioxide against *Cryptosporidium* and *Cyclospora* on lettuce and basil (but not berries) has been tested and the results indicated that it was not effective for *Cyclospora*. In addition, the treatment also resulted in deterioration of the quality of the produce (Ortega et al., 2008).

There are also some indications that different parasites could survive freezing conditions (Barbosa et al., 2012; Jones and Dubey, 2012; O'Lorcain, 1995; Veit et al., 1995). Failing to inactivate parasites on berries that are intended to be used without further treatment may result in human infection, as was seen in a cyclosporiasis outbreak in Philadelphia, U.S. in 2000, where the raspberries used in the filling of a wedding cake was the source of infection (Ho et al., 2002). Although the berries had been frozen, this was insufficient to inactivate the parasites. *Cryptosporidium* oocysts are also resistant to freezing (Fayer and Nerad, 1996).

Developing methods to remove and or inactivate parasites on berries in a way that does not jeopardize the attributes of fresh produce, as well as ensuring consumers' safety should be an area of research focus. It is challenging to preserve the physical properties and nutritional value of the berries and at the same time inactivate or remove parasites. Thermal, chemical, and non-thermal methods used in the range that kills parasite transmission stages all have potentially negative impacts on product quality. Furthermore, methods for decontamination of fresh produce, such as washing ready-to-eat salads in water, have been shown to have the potential to spread focal pathogen contamination of a few products items throughout a whole batch (Gil et al., 2009). This highlights the need for implementing a system for monitoring industrial processing methods.

High-pressure processing (HPP) is one of the more promising technologies that may help overcome these challenges. In a study of raspberries contaminated with *T*. *gondii* oocysts it was shown that HPP treatment at 340 megapascal for 60 s was sufficient to leave oocysts unable to infect mice (Lindsay et al., 2008). Similar results have been found for other parasites and food types, e.g., *C*. *parvum* oocysts in fruit juice (Slifko et al., 2000), and *Eimeria acervulina* (surrogate for *C*. *cayetanensis*) on fresh raspberries and basil leaves (Kniel et al., 2007). However, there is a need for more research that focuses on the effect of HPP on other foodborne parasites and different fresh produce matrices, and also to evaluate whether it can be used effectively on berry products without damaging or degrading their physical properties. Refrigeration might be helpful to delay the infectivity of parasites that require warm temperature for sporulation, e.g., *Cyclospora*, during transport and storage of berries.

Many microorganisms are sensitive to UV light and this type of treatment does not alter the physical properties of most fresh produce products. UV radiation is, therefore, a potentially suitable method to deactivate parasites found on fresh produce. Although several studies have shown sufficient efficacy of UV-radiation on parasite inactivation in water, e.g., for *T*. *gondii*, *Cryptosporidium* spp., and *Giardia* (Campbell and Wallis, 2002; Dumetre et al., 2008; Hayes et al., 2013), only a few studies have investigated whether such treatment is applicable for use on contaminated fresh produce, including berries. Using *Eimeria acervulina* as a surrogate for *C*. *cayetanensis*, (Kniel et al., 2007) found that UV treatment reduced the pathogenic effect of oocysts in chickens, but was not sufficient for complete inactivation of the oocysts. In another study, *C*. *parvum* oocyst infectivity was assessed in immunocompetent suckling mice after pulsed UV irradiation (Le Goff et al., 2015). The oocysts had been spiked onto raspberries that were UV treated with 4 J/cm^2^. Although irradiated oocysts infected fewer mice than non-irradiated oocysts, the UV treatment was not sufficient to inactivate all oocysts. Considering the physical properties of raspberries, it seems probable that some oocysts might have escaped exposure to the UV radiation due to masking in the crevices between the drupelets of the individual berries.

Based on these findings, it is evident that methods for removal or inactivation of parasites on berries are in need of further development. Thus, until new and effective methods are developed that can be implemented in the fresh produce industry, the focus should be on prevention of contamination within the farm-to-fork chain.

## 11 Conclusion

Consumer demand for fresh berries is increasing, resulting in the production and import of greater volumes of berries, and this trend is expected to persist into the foreseeable future. Outbreaks of parasitic diseases associated with consumption of contaminated berries have been reported. Thus, the risk of parasitic infection due to consumption of contaminated berries needs to be addressed. Data on the occurrence of parasites on berries is limited, and appropriate analytical methods have only recently been developed, and only for a very few species of parasites. In order to be able to determine the risk of parasitic infection due to consumption of contaminated berries, developing and improving methods for their detection and conducting appropriate surveys should be considered relevant tasks by those currently profiting from the berry industry. Furthermore, given the current absence of appropriate removal or inactivation measures for parasites that could be implemented in the berry industry, prevention of contamination should be a priority; this should be a part of GAP, GPP, HACCP, and other relevant preventive measures. Thus, control of parasites on fresh berries requires a concerted effort among different stakeholders involved and calls for researchers in the field to cooperate in developing and validating appropriate approaches.
